# MAIT cells are reduced in frequency and functionally impaired in human T lymphotropic virus type 1 infection: Potential clinical implications

**DOI:** 10.1371/journal.pone.0175345

**Published:** 2017-04-06

**Authors:** Dominic Paquin-Proulx, Benjamin C. Greenspun, Emanuela A. S. Costa, Aluisio C. Segurado, Esper G. Kallas, Douglas F. Nixon, Fabio E. Leal

**Affiliations:** 1 Department of Microbiology, Immunology & Tropical Medicine, The George Washington University, Washington, DC, United States of America; 2 Departamento de Moléstias Infecciosas e Parasitárias, Faculdade de Medicina, Universidade de São Paulo, São Paulo, Brasil; University of Cape Town, SOUTH AFRICA

## Abstract

HTLV-1 infection is associated with several inflammatory disorders, including the neurodegenerative condition HTLV-1-associated myelopathy/tropical spastic paraparesis (HAM/TSP). It is unclear why a minority of infected subjects develop HAM/TSP. The cellular immune response has been implicated in the development of inflammatory alterations in these patients; however the pathogenic mechanisms for disease progression remain unclear. Furthermore, HTLV-1-infected individuals have an increase incidence of *Mycobacterium tuberculosis* (Mtb) infection, suggesting that immunological defect are associated with HTLV-1 infection. Evidence suggests an important role for Mucosal-associated invariant T (MAIT) cells in the early control of Mtb infection. Chronic viral infections like HIV and HCV have been associated with decreased frequency and functionality of MAIT cells. We hypothesized that HTLV-1 infection is associated with similar perturbations in MAIT cells. We investigated MAIT cell frequency, phenotype, and function by flow cytometry in a cohort of 10 asymptomatic and 10 HAM/TSP HTLV-1 infected patients. We found that MAIT cells from HTLV-1-infected subjects were reduced and showed high co-expression of the activation markers CD38 and HLA-DR but normal levels of CCR6 and CD127. MAIT cells had a lower expression of the transcription factor PLZF in HAM/TSP patients. Unlike Tax-specific CD8+T cells, which are hyperfunctional, MAIT cells from HTLV-1-infected subjects had a poor IFNγ response following antigen stimulation. MAIT cell perturbations in HTLV-1 infection were not associated with HTLV-1 proviral load and MAIT cells were not infected by HTLV-1 *in vivo*. Rather, MAIT cells loss was associated with immune activation. Overall, our results do not support a role for MAIT cells in HAM/TSP pathogenesis but reduced numbers of MAIT cells, together with their poor functionality, could contribute to the increased susceptibility of HTLV-1-infected individuals to other infectious agents.

## Introduction

Human T-lymphotropic virus type 1 (HTLV-1) is a deltaretrovirus estimated to infect around 10 million people worldwide[1], with no clinical evidence of infection in the majority of carriers. The life-long risk of developing HTLV-1-associated pathology is approximately 10%, while 0.3 to 4% of HTLV-1 carriers will present a neurodegenerative disorder with clinical similarities to Multiple Sclerosis (MS) called HTLV-1-Associated Myelopathy/Tropical Spastic Paraparesis (HAM/TSP)[2]. Other inflammatory autoimmune-like conditions including Sjögren syndrome, uveitis and arthritis have been reported to be associated with HTLV-1 infection[3–6]. HTLV-1 is also the causative agent of an aggressive type of lymphoproliferative disorder called Adult T-cell leukemia[7]. HTLV-1-infected subjects have also an increased susceptibility to *Mycobacterium tuberculosis* (Mtb) infection[8, 9], suggesting an immunological impairment[10]. The role of the various T-cell subsets in the immune response to infection HTLV-1 and how it influences the control or the development of disease is not fully understood.

CD4+ T cells are the primary targets of HTLV-1 *in vivo*[11]. The immunodominant viral proteins Tax and HBZ modulate CD4 T-cell machinery upon infection, promoting cell activation, replication and survival[12, 13]. The high frequency of HTLV-1-specific IFN-γ-producing CD4+ and CD8+ T cells in the central nervous system (CNS) and in peripheral blood seen in HAM/TSP patients suggest that T-cell responses do not prevent development of disease, contributing instead to the proinflammatory milieu characteristic of HTLV-1 related syndromes[14–16]. Other T-cell subsets such as NKT cells were investigated in the context of HTLV-1 infection[17], but many aspects of the immunopathogenesis of HTLV-1 related diseases and why HTLV-1 patients are more susceptible to Mtb are still unexplored.

Mucosal-associated invariant T (MAIT) cells are a population of innate T cells recently identified. In human, they express a semi-invariant TCR using Vα7.2 coupled with Jα33 and have a limited Vβ repertoire[18]. They can represent 1–10% of circulating T cells in healthy individuals. They are restricted by the MHC class I-like protein MR1 that presents microbial vitamin B2 metabolites (riboflavin)[19]. MAIT cells have been shown to recognize a variety of pathogens, including Mtb *in vitro* and *in vivo*[20, 21]. MAIT cells can also be activated indirectly by IL-12 and IL18[22]. MAIT cells have been implicated in the immunopathogenesis of chronic viral infections like HIV[23, 24] and HCV[25, 26] and in inflammatory conditions like systemic lupus erythematosus[27] and MS[28, 29]. Therefore, we hypothesized that HTLV-1 infection is associated with defect in MAIT cells that could explain the increased susceptibility to Mtb.

In this study, we determined the frequency, phenotype, and functionality of MAIT cells in a cohort of HTLV-1-infected subjects. Here, we describe for the first time phenotypic and functional alterations in MAIT cells of HTLV-1-infected individuals and discuss the implications such alterations might have in HAM/TSP and in the immune response of HTLV-1-infected subjects to other pathogens, specifically Mtb.

## Materials and methods

### Human subjects

We selected 20 HTLV-1 patients from the HTLV-1 outpatient clinic at the University of Sao Paulo, Brazil. All participants were enrolled in a longitudinal cohort of HTLV-1-infected subjects after signing a written consent form approved by the University of São Paulo’s Institutional Review Board (#0855/08). Patients were recruited and peripheral blood was collected between October 2014 and October 2015. Patients were invited to participate in the study based on the following criteria: capacity to understand and sign consent form, age > 18, HIV negative, no therapy with corticosteroids or immunomodulators in the last 3 months and absence of active co-infection by Tuberculosis or Syphilis. We randomly selected 10 patients diagnosed with neurological complications associated to HTLV-1 infection (HAM/TSP), 10 HTLV-1 asymptomatic carriers and 12 uninfected controls age-and-gender-matched (Table 1). Clinical status was determined by the World Health Organization criteria for HTLV-1 associated diseases[30]. Neither the Kurtze Expanded Disability Status Scale (EDSS) nor other methods to quantify motor disability were available for this cohort. There was no significant difference in gender (chi-square p-value = 0.88) and age between the groups. HTLV-1 proviral load was significantly higher in HAM/TSP compared to asymptomatic subjects (2.15 vs 10.56; p<0.01; Table 1).

**Table 1:** Patients demographics.

	Number	Gender	Age	Proviral load
			Median (range):	
Controls	12	3M 9F	51.5 (27–69)	
			Individual:	
	1	M	28	
	2	M	43	
	3	M	68	
	4	F	52	
	5	F	41	
	6	F	60	
	7	F	51	
	8	F	38	
	9	F	27	
	10	F	69	
	11	F	55	
	12	F	53	
			Median (range):	Median (interquartile):
Asymptomatic	10	3M 7F	53.5 (38–75)	2.15 (1.14;3.92)
			Individual:	Individual:
	1	M	72	4.01
	2	M	75	1.64
	3	M	53	0.38
	4	F	68	2.50
	5	F	47	7.19
	6	F	67	0.17
	7	F	50	1.79
	8	F	52	0.98
	9	F	38	3.62
	10	F	69	9.64
			Median (range):	Median (interquartile):
			54 (27–65)	10.56 (7.57;14.67)
HAM/TSP	10	2M 8F	Individual:	Individual:
	1	M	53	14.56
	2	M	27	11.59
	3	F	65	7.3
	4	F	62	48.28
	5	F	47	14.71
	6	F	52	44.06
	7	F	61	9.53
	8	F	55	8.37
	9	F	56	0.19
	10	F	21	3.70

### Flow cytometry and monoclonal antibodies

Cryopreserved PBMC specimens were thawed and washed, and counts and viability were assessed using the Countless Automated Cell Counter system (Invitrogen, Carlsbad, CA). Cells were washed and stained in Brilliant Violet Stain Buffer (BD Biosciences, San Jose, CA) at room temperature for 15 min in 96-well V-bottom plates in the dark. Samples were then washed and fixed using Cytofix/Cytoperm (BD Biosciences) before flow cytometry data acquisition. Intracellular staining for IFNγ and promyelocytic leukaemia zinc finger (PLZF) were performed in Perm/Wash (BD Biosciences) at room temperature for 15 min in the dark. mAbs used in flow cytometry: CD3 AF700, CD3 PerCP-Cy5.5 (both clone UCHT1), CD4 BV605 (clone RPA-T4), CD8 BV711 (clone RPA-T8), CD38 APC-H7 (clone HB7), CD69 AF00 (clone FN50de), CD161 BV421 (clone DX12), CCR6 BV786 (clone 11A9), HLA-DR APC (clone L243), IFNγ APC (clone B27), and PD-1 PE-Cy7 (clone EH12.1) were all from BD Biosciences, TCR Vα7.2 PE, TCR Vα7.2 PerCP-Cy5.5 (clone 3C10) were from Biolegend (San Diego, CA, USA), and PLZF APC was from R&D Systems (Minneapolis, MN). Live/dead aqua fixable cell stain was from Life Technologies (Eugene, OR, USA) and added to the cells together with the cell surface antibodies. Data were acquired on a BD LSRFortessa instrument (BD Biosciences) and analyzed using FlowJo Version 9.8.5 software (TreeStar, Ashland, OR, USA).

### Functional assay

MAIT cell function was determined *in vitro* using paraformaldehyde-fixed *E*. *coli* stimulation (one shot top10, Life Technology, MOI 10) in the presence of 1.25 μg/ml anti-CD28 mAb (clone L293, BD Biosciences)[31]. PBMCs were further cultured for 24 hours at 37°C/5% CO_2_ in RPMI medium supplemented with 10% fetal bovin serum (FBS). Monensin (Golgi Stop, BD Biosciences) was added during the last 6 hours of the stimulation.

### Cell sorting

PBMC samples were thawed as previously described and stained with CD3 PerCP-Cy5.5, CD4 APC (clone RTA-T4, BD Bioscience), CD161 FITC (clone DX12, BD Bioscience), Vα7.2 PE, and DAPI (BD Bioscience) for 10 minutes at RT and washed with PBS 2% FBS. CD4+ T cells and CD4- MAIT cells were then sorted on a SH800Z (Sony Biotechnology, San Jose, CA). Purity was typically over 90%. Sorted cells were then used for pro-viral quantification.

### Pro-viral load quantification

Total DNA was extracted from PBMCs using a commercial kit (Qiagen GmbH, Hilden Germany) following the manufacturer's instructions. For HTLV-1 proviral load absolute quantification, a double standard plasmid pcHTLV-ALB previously described was used to generate standard curves[32]. For each run, standard curves for the value of plasmid pcHTLV-ALB were generated of log_10_ dilutions (from 10^5^ to 10^0^ copies). For quantitation of HTLV-1 and the human housekeeping gene albumin in genomic DNA, previously described primers combined with TaqMan^**®**^ Universal Master Mix II (ThermoFisher Scientific) were used[32]. Each sample was assayed in duplicate and the mean of the two values was considered as the copy number of the sample. The amount of HTLV-1 pro-viral load was calculated as follows: copy number of HTLV-1 per 1,000 PBMCs = (copy number of HTLV-1)/(copy number of albumin) x 2 x 1000 cells.

### Statistical analysis

All statistical analysis was performed using Graph Pad Prism version 6.0 for Mac OSX (GraphPad Software, La Jolla, CA). The comparison between healthy controls and HTLV-1 patients and between asymptomatic and HAM/TSP patients were analyzed using Mann Whitney U-test. Associations between groups were determined by Spearman's rank correlation. P values **≤** 0.05 were considered statistically significant.

## Results

### MAIT cells are reduced in HTLV-1 infection and show increased expression of activation markers

**Fig 1:**
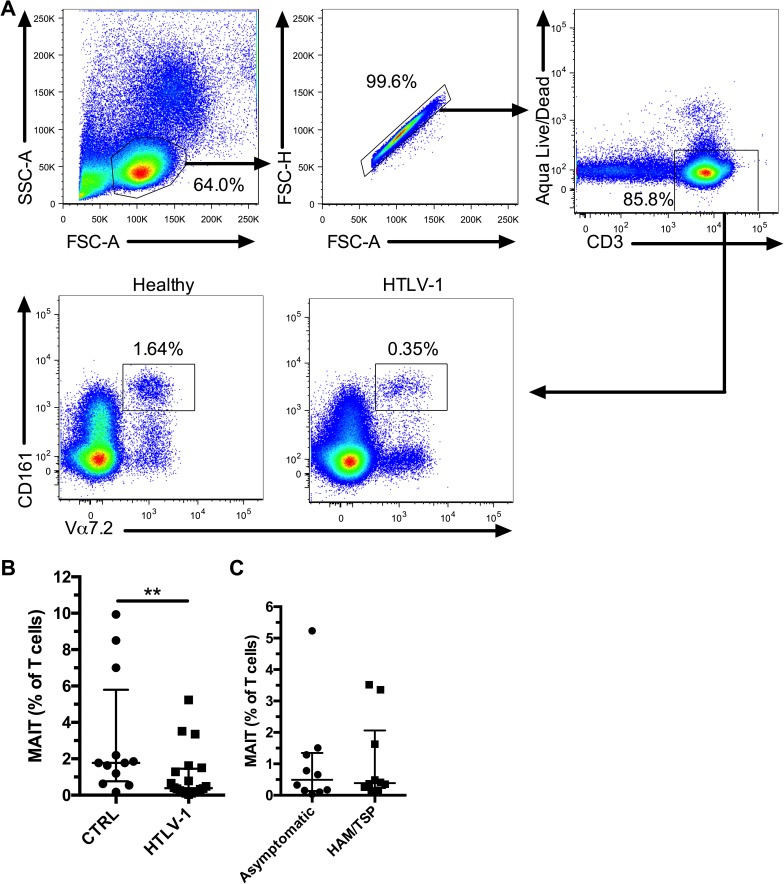
MAIT cells are reduced in HTLV-1 infection. Representative flow plots showing gating strategy and MAIT cell frequency in healthy controls and HTLV-infected individuals (A). Frequency of MAIT cells in healthy controls (n = 12) and HLTV-1 patients (n = 20) (B). Frequency of MAIT cells in asymptomatic (n = 10) and HAM/TSP (n = 10) HTLV-1 patients (C). ** indicates p < 0.001. The lines and whiskers represent the median and interquartile range respectively.

First, we evaluated the frequency of MAIT cells by flow cytometry in cryopreserved PBMCs from 20 HTLV-1-infected individuals (10 asymptomatic carriers and 10 with HAM/TSP) and in 12 matched healthy controls (Table 1). MAIT cells were found to be significantly reduced in HTLV-1 patients (Fig 1A and 1B). We did not observe any difference in the MAIT cell frequency between asymptomatic carriers and HAM/TSP patients (Fig 1C).

**Fig 2:**
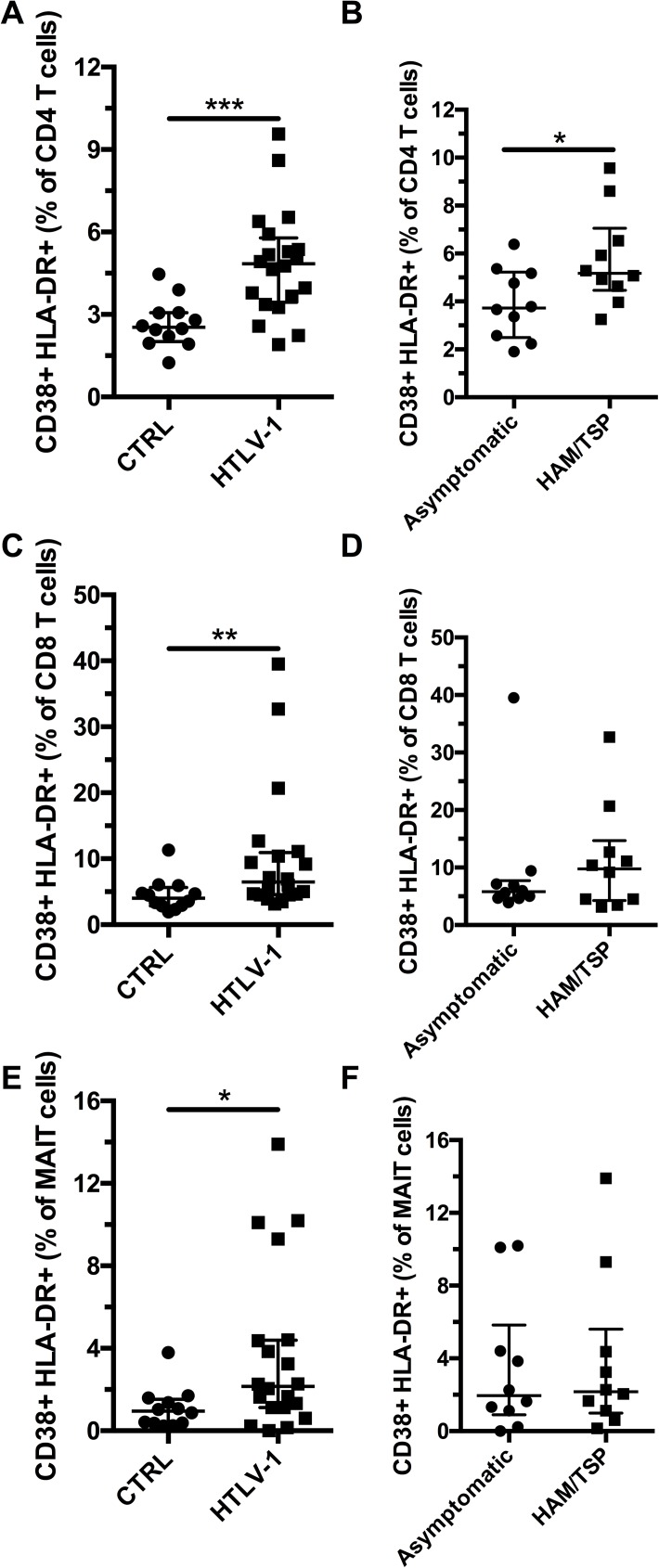
MAIT cells are activated in HTLV-1 infection. Co-expression of CD38 and HLA-DR by CD4 T cells in healthy controls (n = 12) and HLTV-1 patients (n = 20) (A). Co-expression of CD38 and HLA-DR by CD4 T cells in asymptomatic (n = 10) and HAM/TSP (n = 10) HTLV-1 patients (B). Co-expression of CD38 and HLA-DR by CD8 T cells in healthy controls and HLTV-1 patients (C). Co-expression of CD38 and HLA-DR by CD4 T cells in asymptomatic and HAM/TSP HTLV-1 patients (D). Co-expression of CD38 and HLA-DR by MAIT cells in healthy controls and HLTV-1 patients (E). Co-expression of CD38 and HLA-DR by MAIT cells in asymptomatic and HAM/TSP HTLV-1 patients (F). * indicates p **≤** 0.05, ** indicates p < 0.01, and *** indicates p < 0.001. The lines and whiskers represent the median and interquartile range respectively.

Next, we studied the expression of the activation markers CD38 and HLA-DR by CD4 T cells, CD8 T cells, and MAIT cells (S1 Fig). CD4 T cells showed an increase in co-expression of CD38 and HLA-DR in HTLV-1-infected subjects when compared to uninfected individuals (Fig 2A). HAM/TSP patients had higher CD4 T cell activation compared to asymptomatic carriers (p = 0.05, Fig 2B). Elevated co-expression of CD38 and HLA-DR was also found on CD8 T cells of HTLV-1-infected individuals but no difference was observed between asymptomatic carriers and HAM/TSP patients (Fig 2C and 2D). We observed elevated levels of activation markers (CD38 and HLA-DR) on MAIT cells from HTLV-1-infected patients but no significant difference was observed between asymptomatic carriers and uninfected individuals (Fig 2E and 2F). We also evaluated the expression of the exhaustion marker PD-1 and did not observe any significant difference between controls and HTLV-1-infected subjects or between asymptomatic carriers and HAM/TSP patients (S2 Fig). Expression of PD-1 by MAIT cells showed a trend to associate with their expression of activation markers (p = 0.0686, S2 Fig). Our results show that HTLV-1 patients have a significantly lower frequency of MAIT cells and that residual MAIT cells have increased levels of expression of activation markers.

### Reduced frequency of MAIT cells correlates with increased expression of activation markers but not with HTLV-1 proviral load

**Fig 3:**
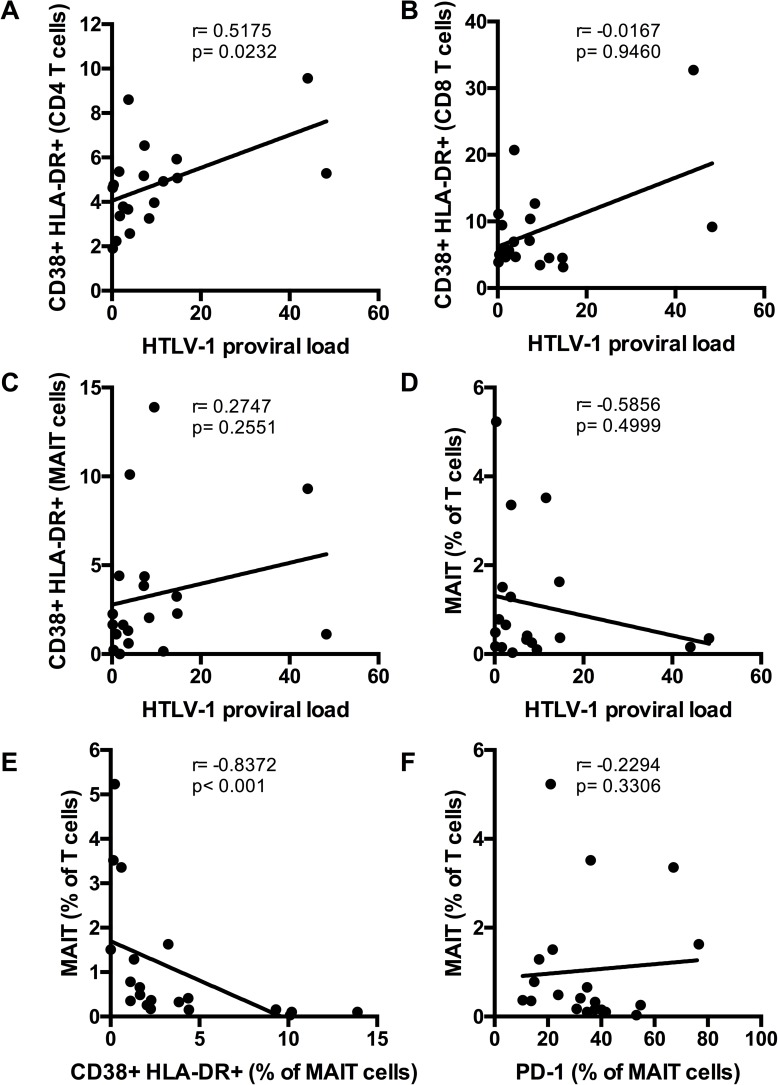
Associations between immune activation and HTLV-1 proviral load. Associations between proviral load and CD4 T cell (A), CD8 T cell (B), and MAIT cell (C) co-expression of CD38 and HLA-DR. Association between proviral load and MAIT cell frequency (D). Association between co-expression of CD38 and HLA-DR (E) and PD-1 expression (F) by MAIT cells and MAIT cell frequency.

We then looked for associations between immune activation and HTLV-1 proviral load. As expected, we found a positive association between HTLV-1 proviral load and CD4 T cell activation (r = 0.5175, p = 0.0232, Fig 3A) but not with CD8 T cell activation (p = 0.9460, Fig 3B) or with MAIT cell activation (p = 0.2551, Fig 3C). HTLV-1 proviral load was also not associated with MAIT cell frequency (p = 0.4999, Fig 3D). However, MAIT cell frequency was inversely associated with their co-expression of CD38 and HLA-DR (p<0.001, Fig 3E) but not with PD-1 expression (p = 0.3306, Fig 3F). This suggests that immune activation associated with HTLV-1 infection results in loss of MAIT cells.

### MAIT cells are not infected by HTLV-1 *in vivo*

**Fig 4:**
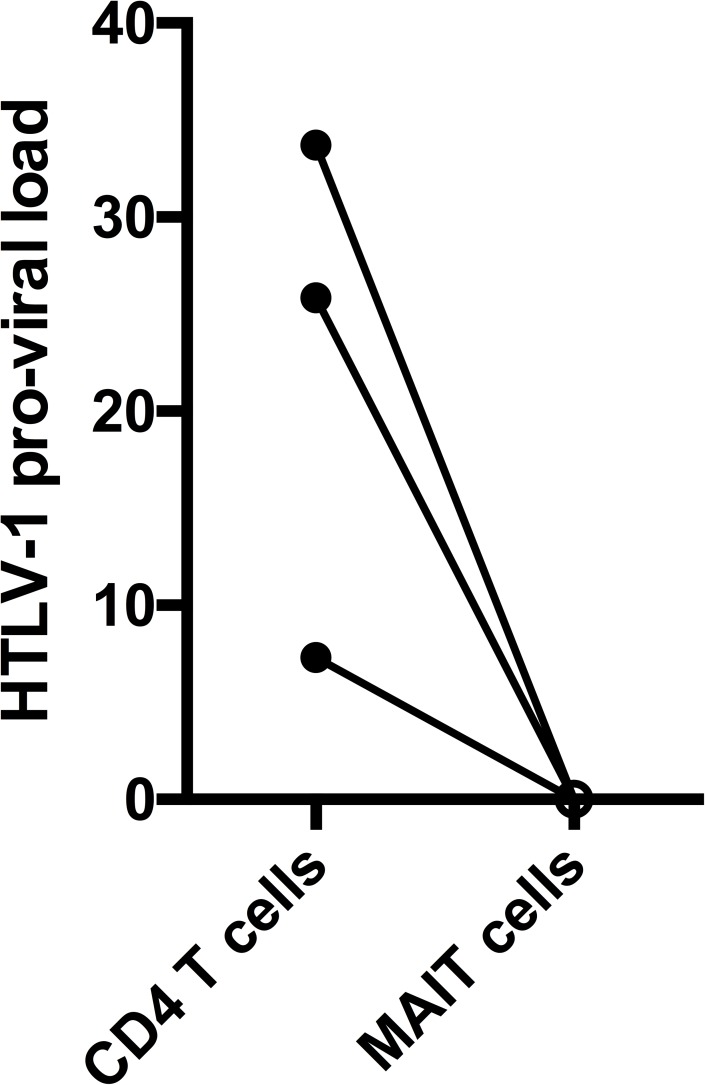
MAIT cells are not preferentially infected by HTLV-1. Proviral load in sorted CD4 T cells (closed circles) and MAIT cells (open circles) from three HTLV-1-infected subjects.

To investigate the capacity of HTLV-1 to infect MAIT cells in vivo, we sorted CD4 T cells and MAIT cells from 3 HTLV-infected subjects and measured the pro-viral load in the sorted cells. We found that the HTLV-1 pro-viral load in MAIT cells was below detection limit in all 3 individuals (Fig 4). In contrast, HTLV-1 pro-viral load was detected in CD4 T cells from all subjects.

### Lower PLZF expression by MAIT cells in HAM/TSP patients

**Fig 5:**
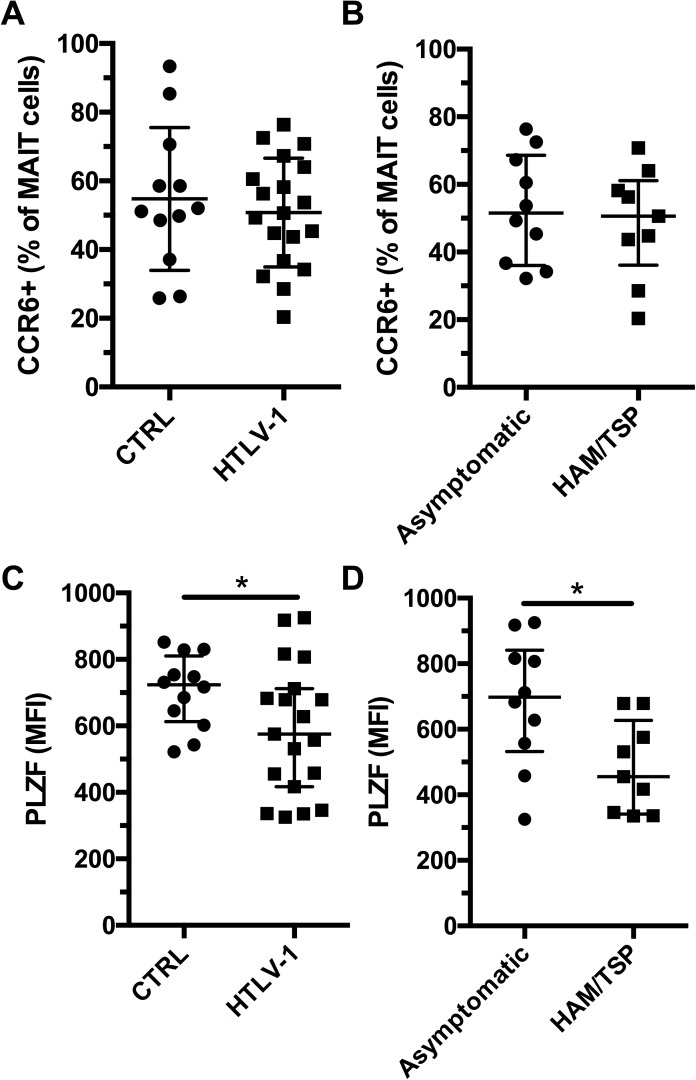
Loss of PLZF expression by MAIT cells in HAM/TSP patients. Expression of CCR6 by MAIT cells (CD3+ Vα7.2+ CD161+) in healthy controls (n = 12) and HTLV-1infected subjects (n = 19) (A). Expression of CCR6 by MAIT cells in asymptomatic (n = 10), and HAM/TSP (n = 9) HTLV-1-infected subjects (B). PLZF expression (MFI) by MAIT cells from healthy controls and HTLV-1-infected individuals (C). PLZF expression (MFI) by MAIT cells in asymptomatic, and HAM/TSP HTLV-1-infected subjects (D). * indicates p **≤** 0.05. The lines and whiskers represent the median and interquartile range respectively.

In normal conditions, MAIT cells express CCR6 and the transcription factor PLZF[33, 34]. PLZF expression by MAIT is essential for their effector functions[35]. However, it has been reported that expression of CCR6 and PLZF is decreased in a number in viral infections[36–39]. Thus, we evaluated the expression levels of CCR6 and PLZF by MAIT cells in the HTLV-1 cohort. We found a similar frequency of CCR6+ MAIT cells in HTLV-1 infection compared to controls and no difference between asymptomatic and HAM/TSP patients (Fig 5A and 5B and S3 Fig). However, MAIT cells from HTLV-1 patients had significantly lower levels of PLZF compared to healthy controls (Fig 5C and S3 Fig). MAIT cells from HAM/TSP patients had significantly lower levels of PLZF than asymptomatic carriers (Fig 5D). This results suggest that residual MAIT cells in HTLV-1 infection might be functionally impaired.

### MAIT cell response to E. coli stimulation is reduced in HTLV-1 infection

**Fig 6:**
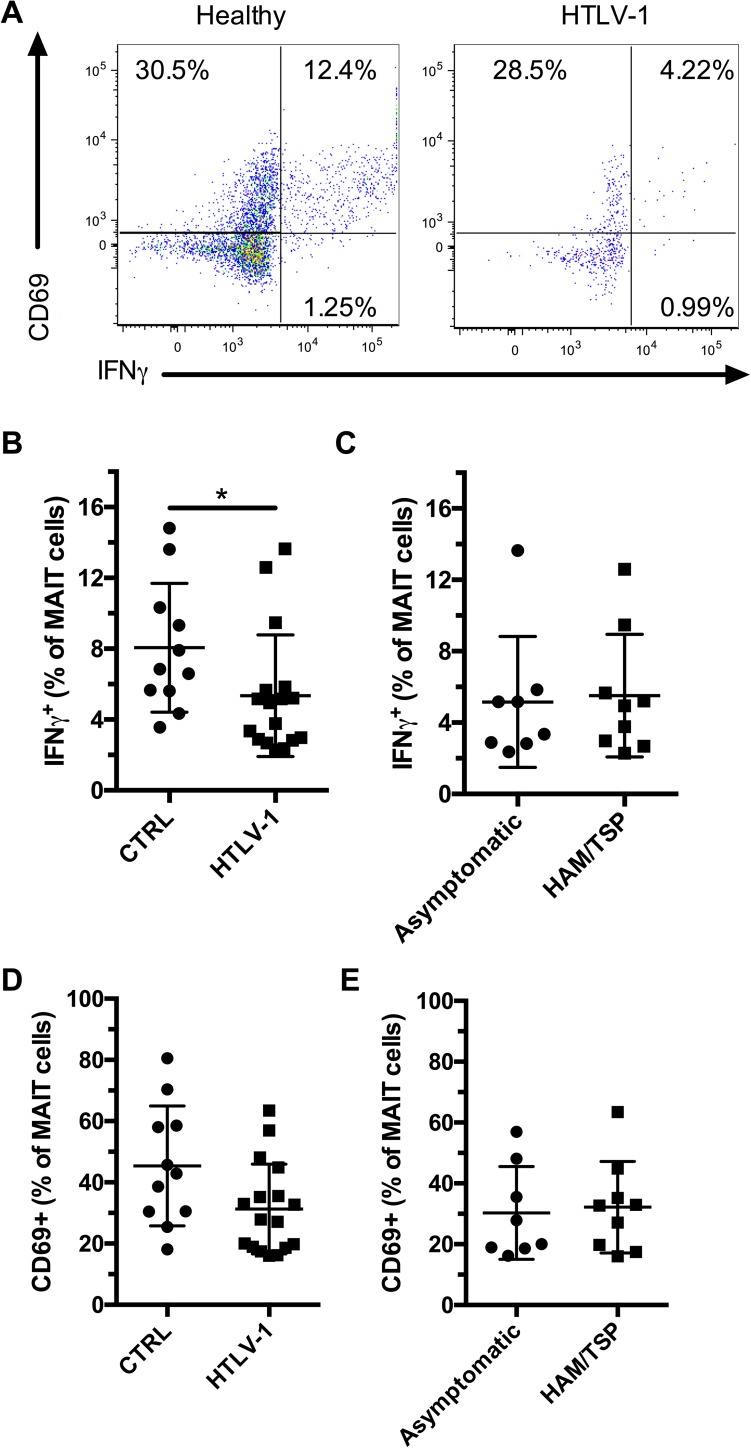
MAIT cells are functionally impaired in HTLV-1 infection. PBMCs were stimulated with *E*. *coli* for 24h at a MOI of 10 and Monensin was added during the last 6 hours before staining for surface antigens and intracellular staining for IFNγ. Representative flow plots of CD69 expression and IFNγ production by MAIT cells (CD3+ Vα7.2+ CD161+) from healthy controls and HTLV-1-infected individuals (A). Production of IFNγ by MAIT cells in healthy controls (n = 11) and HTLV-1-infected subjects (n = 17) (B). Production of IFNγ by MAIT cells in asymptomatic (n = 8), and HAM/TSP (n = 9) HTLV-1-infected subjects (C). CD69 expression by MAIT cells in healthy controls and HTLV-1-infected subjects (D). CD69 expression by MAIT cells in asymptomatic, and HAM/TSP HTLV-1-infected subjects (E). * indicates p **≤** 0.05 and ** indicates p < 0.01. The lines and whiskers represent the median and interquartile range respectively.

Finally, we stimulated PBMCs from healthy controls and HTLV-1-infected subjects with fixed *E*. *coli* to stimulate MAIT cells and evaluated their production of IFNγ and up-regulation of CD69 (Fig 6A). There was no difference in spontaneous IFNγ production by MAIT cells in unstimulated samples between the groups (S4 Fig). IFNγ production following *E*. *coli* stimulation was lower in HTLV-1-infected individuals compared to healthy controls (Fig 6B), and there was no difference between asymptomatic carriers and HAM/TSP patients (Fig 6C). We observed a non-significant trend for a reduction in CD69 up-regulation in the HTLV-1-infected subjects compared to controls and no difference between asymptomatic carriers and HAM/TSP patients (Fig 6D and 6E). Our results show that HTLV-1 infection is associated with a poor MAIT cell IFNγ response following bacterial stimulation.

## Discussion

In this study, we found that MAIT cells are reduced in HTLV-1-infected subjects and that the residual MAIT cells display an activated phenotype with a poor IFNγ response following stimulation with *E*. *coli*. We did not observe any difference in frequency of MAIT cells between asymptomatic carriers and HAM/TSP patients. This is in contrast with what has been reported for iNKT cells, another subset of innate T cells, that were further reduced in HAM/TSP patients[17, 40].

Our results reproduce what has been described in other chronic viral infections. A similar loss of MAIT cells with residual MAIT cells showing an activated phenotype and loss of PLZF expression has been described in HIV and HCV infections[23–26]. None of the MAIT cells alterations that we report here were associated with the HTLV-1 proviral load and MAIT cells were not found to be infected by HTLV-1 *in vivo*. Rather, MAIT cell loss was found to be associated with their activation status. Thus, our results suggest that the inflammatory state induced by HTLV-1 infection could be responsible for the MAIT cell loss. Recently, MAIT cells have been reported to respond to virus-infected cells in a TCR-independent way[41]. Virus-induced MAIT cell activation was dependent on the IL-18 pathway and could synergize with IL-12, IL-15 and IFNα/β. Little is known regarding IL-18 levels in HTLV-1 infection but polymorphisms in the IL-18 promoter have been associated with susceptibility to infection[42]. IL-12 secretion by neutrophils was found to be elevated in asymptomatic and symptomatic HTLV-1 carriers[43]. IL-12 was also found to be elevated in HTLV-1 patients with periodontitis compared with patients with periodontitis only[44] and in HTLV-1 patients with HAM/TSP compared to uninfected patients with neurological diseases[45]. A type I interferon-induced gene signature has been identified in individuals with HAM/TSP but abnormal plasma levels of type I or type II IFNs haven’t been detected in those patients, probably because of the short half-lives of IFNs[46]. Elevated levels of IL-12, IL-18, and IFNα/β in HTLV-1 infection could explain the activated MAIT cell phenotype that we observed. Chronic *in vivo* stimulation could also lead to exhaustion and explain the poor *in vitro* IFNγ response following *E*. *coli* stimulation that we observed.

HTLV-1-infected subjects have about a 3-fold increased susceptibility to Mtb than non-infected individuals, irrespective of their HIV status[8]. Interestingly, there was no difference in the Mtb clinical outcome between HTLV-1-infected individuals and controls[8], suggesting that early innate response against Mtb is defective in HTLV-1 infection. MAIT cells are capable of recognizing Mtb-infected cells[20] and MAIT cells have been shown in a mice model to contribute to the early protection against Mtb infection[21]. MAIT cells have also been shown to respond to both Bacillus Calmette-Guerin vaccination and Mtb infection in a non-human primate model[47]. Therefore, the reduced frequency and functionality of MAIT cells that we observed in HTLV-1-infected subject could contribute to their increased susceptibility to Mtb. It would be important to determine if lung-resident MAIT cells are also decreased and functionally impaired in HTLV-1 infection. Elevated levels of IFNα/β is a known correlate of risk progression for Mtb[48, 49]. In addition to modulating phagocytes[50], IFNα/β could contribute to the increased susceptibility to Mtb of HTLV-1 patients by acting on MAIT cells[41].

In MS, MAIT cells have been postulated to be recruited from the blood to the CNS where they could be activated via a MR1 and IL-18 dependent pathway and contribute to the pathology[29]. The reduced frequency of circulating MAIT cells in HTLV-1-infected subjects and their high level of immune activation could be consistent with recruitment to the CNS. However, we did not observe differences in MAIT cells frequency and activation between asymptomatic carriers and HAM/TSP patients. More studies looking at recruitment of MAIT cells to the CNS are needed to determine the contribution of MAIT cells to HAM/TSP. Other limitations of our study are that it is not possible to determine if MAIT cells are reduced because of recruitment to tissues or of a permanent loss and the polyfunctionality of the residual MAIT cells, including killing capacity, was not addressed.

Altogether, our results suggest that chronic inflammation in HTLV-1 infection leads to activation and loss of MAIT cells. Our results also argue against a role for MAIT in development of neurological symptoms in a subset of infected individuals but could partially explain their higher susceptibility to Mtb.

## Supporting information

S1 Fig CD38 and HLA-DR co-expression in HTLV-1 infection. Representative flow plots of CD38 and HLA-DR co-expression for CD4 T cells, CD8 T cells and MAIT cells in healthy controls and HTLV-1-infected individuals.
(TIFF)

S2 Fig PD-1 expression in HTLV-1 infection. Representative flow plots showing PD-1 expression by MAIT cells (CD3+, Vα7.2+, CD161+) from healthy controls and HTLV-1-infected subjects (A). PD-1 expression by CD4 T cells (B), CD8 T cells (C), and MAIT cells (D) from healthy controls (n = 12), asymptomatic carriers (n = 10), and HAM/TSP patients (n = 10). Association between co expression of CD38 and HLA-DR by MAIT cells and PD-1 expression by MAIT cells (E).
(TIFF)

S3 Fig CCR6 and PLZF expression by MAIT cells in HTLV-1 infection. Representative flow plots showing CCR6 expression by MAIT cells (CD3+, Vα7.2+, CD161+) from healthy controls and HTLV-1-infected subjects (A). Representative flow plots showing PLZF expression in healthy controls, asymptomatic carriers, and HAM/TSP patients (B).
(TIFF)

S4 Fig IFNγ production by unstimulated MAIT cells. IFNγ production by unstimulated MAIT cells from healthy controls (n = 11) and HTLV-1-infected subjects (n = 17) (A). IFNγ production by unstimulated MAIT cells from asymptomatic carriers (n = 8), and HAM/TSP patients (n = 9) (B).
(TIFF)

## Abbreviations

HTLV: Human T-lymphotropic virus
HAM/TSP: HTLV-1-associated myelopathy/tropical spastic paraparesis
MS: multiple sclerosis
Mtb: *Mycobacterium Tuberculosis*
CNS: central nervous system
MAIT: mucosal-associated invariant T
PLZF: promyelocytic leukaemia zinc finger
