# Supplementary figures and images for: MAIT cells are reduced in frequency and functionally impaired in human T lymphotropic virus type 1 infection: Potential clinical implications
Source: PLoS One. 2017 Apr 6;12(4):e0175345. doi: 10.1371/journal.pone.0175345 (PMC5383303; doi:10.1371/journal.pone.0175345)

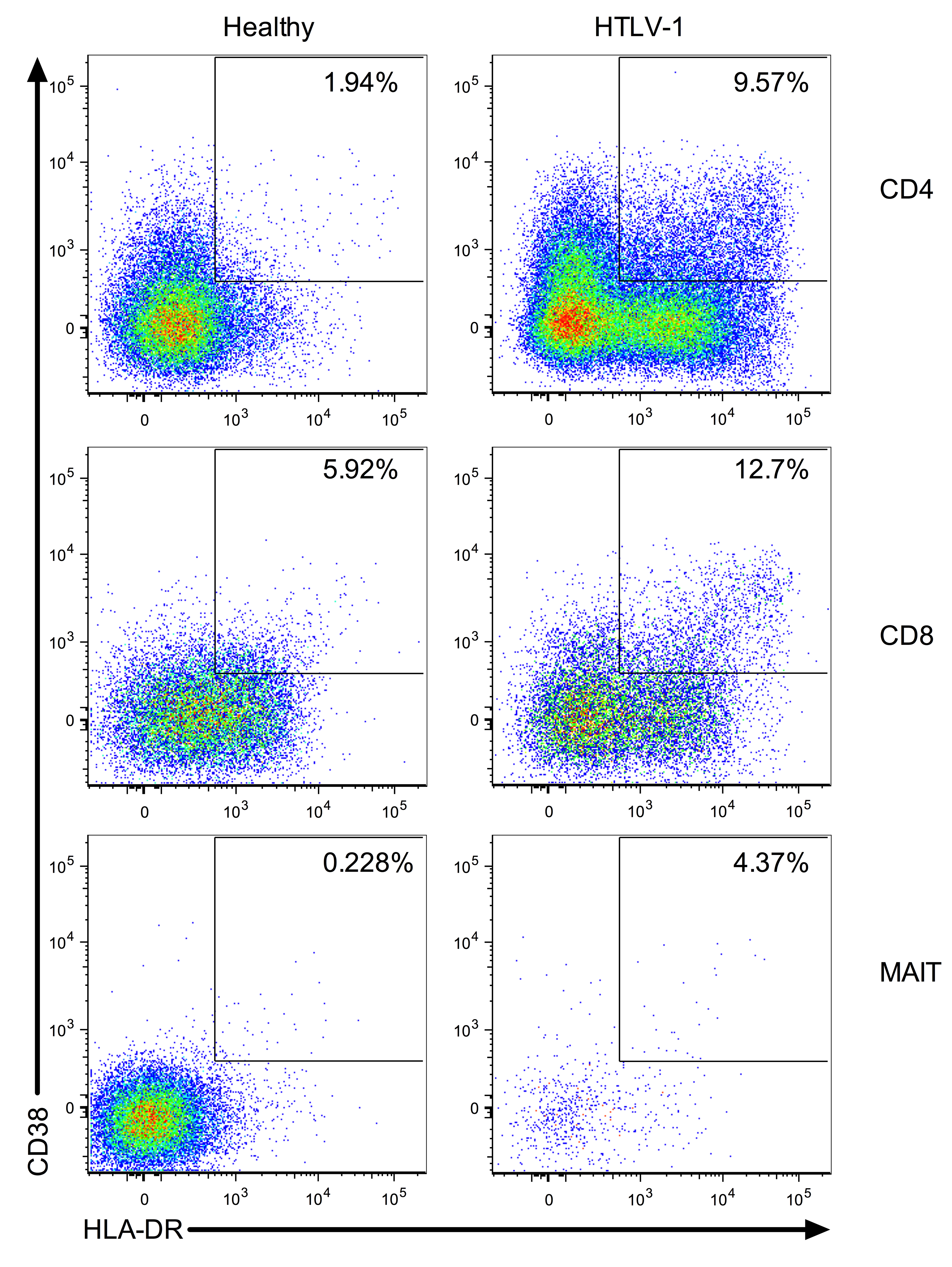

Supplement: S1 Fig — Representative flow plots of CD38 and HLA-DR co-expression for CD4 T cells, CD8 T cells and MAIT cells in healthy controls and HTLV-1-infected individuals. (TIFF) [file pone.0175345.s001.tiff]

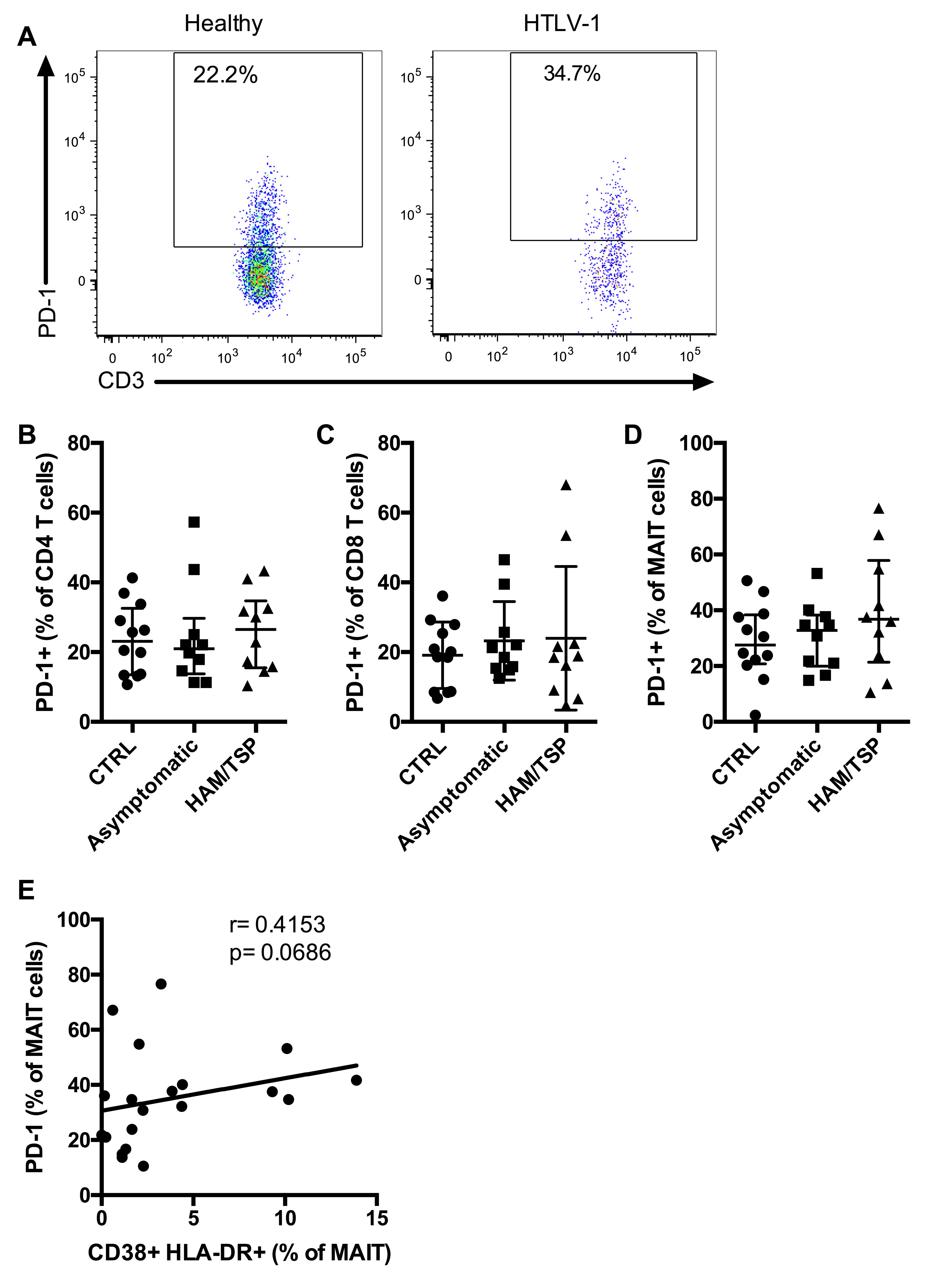

Supplement: S2 Fig — Representative flow plots showing PD-1 expression by MAIT cells (CD3+, Vα7.2+, CD161+) from healthy controls and HTLV-1-infected subjects (A). PD-1 expression by CD4 T cells (B), CD8 T cells (C), and MAIT cells (D) from healthy controls (n = 12), asymptomatic carriers (n = 10), and HAM/TSP patients (n = 10). Association between co expression of CD38 and HLA-DR by MAIT cells and PD-1 expression by MAIT cells (E). (TIFF) [file pone.0175345.s002.tiff]

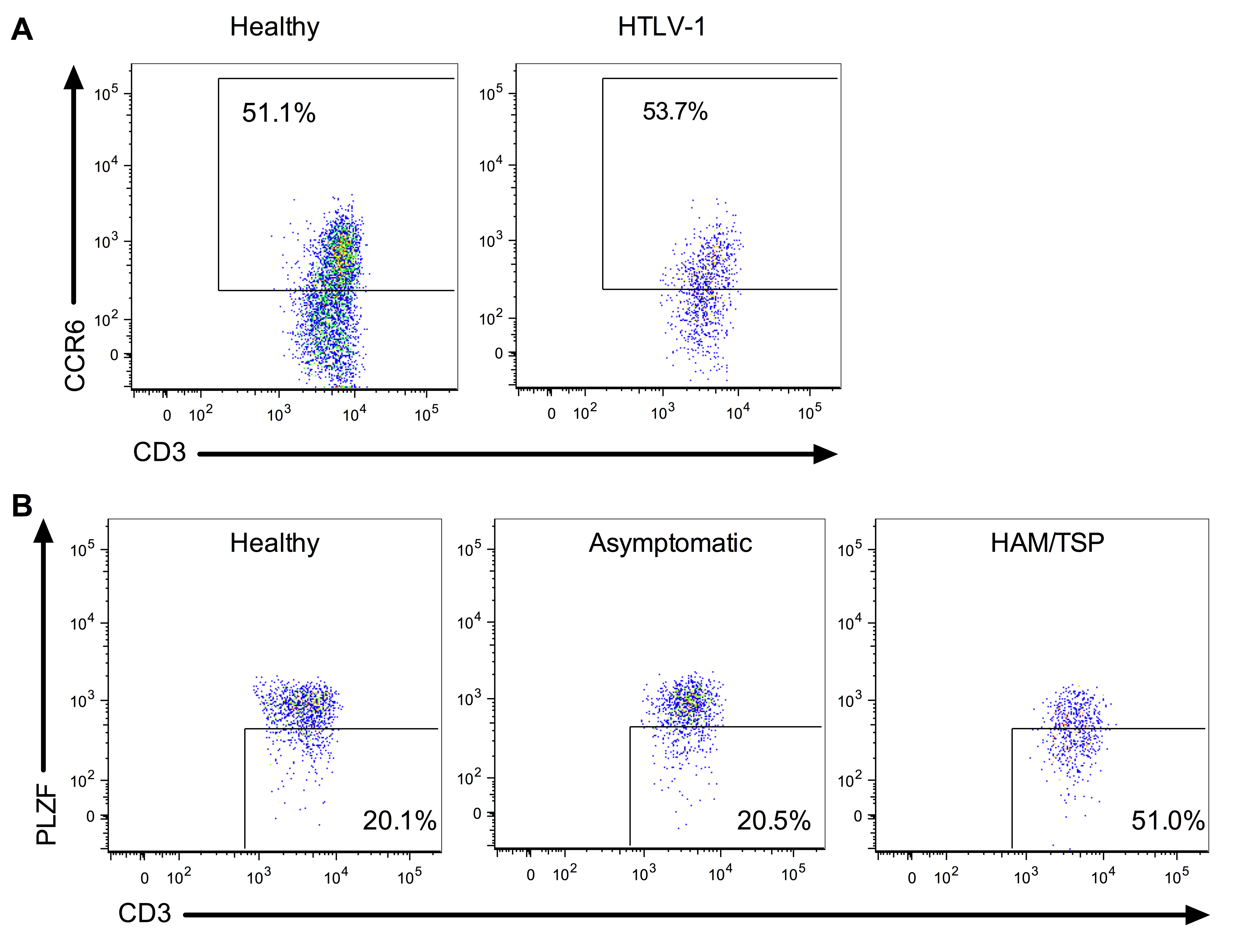

Supplement: S3 Fig — Representative flow plots showing CCR6 expression by MAIT cells (CD3+, Vα7.2+, CD161+) from healthy controls and HTLV-1-infected subjects (A). Representative flow plots showing PLZF expression in healthy controls, asymptomatic carriers, and HAM/TSP patients (B). (TIFF) [file pone.0175345.s003.tiff]

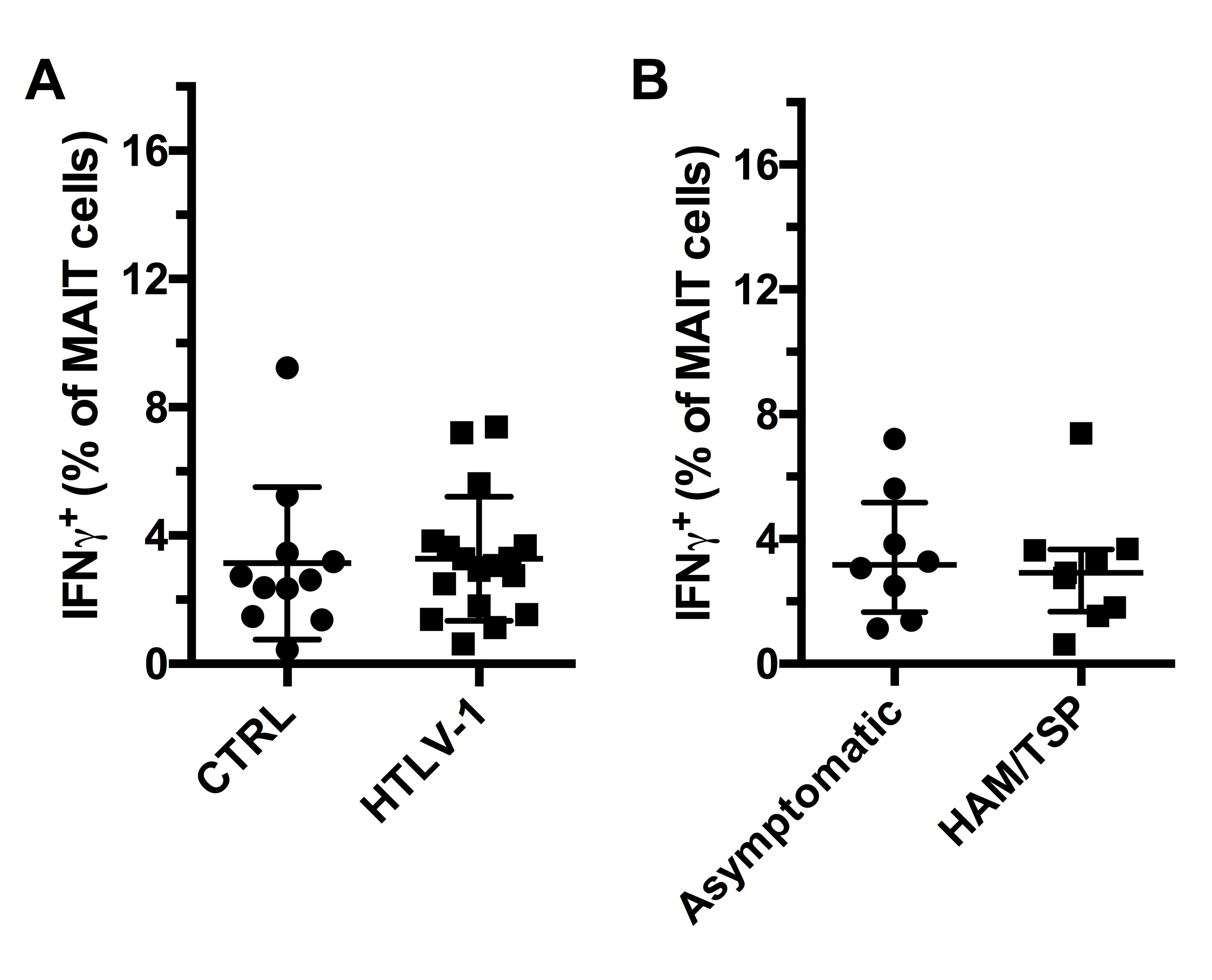

Supplement: S4 Fig — IFNγ production by unstimulated MAIT cells from healthy controls (n = 11) and HTLV-1-infected subjects (n = 17) (A). IFNγ production by unstimulated MAIT cells from asymptomatic carriers (n = 8), and HAM/TSP patients (n = 9) (B). (TIFF) [file pone.0175345.s004.tiff]
